# Modeling the growth of *Staphylococcus aureus* as affected by black zira (*Bunium persicum*) essential oil, temperature, pH and inoculum levels

**Published:** 2014

**Authors:** Abdollah Jamshidi, Saeid Khanzadi, Majid Azizi, Mohammad Azizzadeh, Mohammad Hashemi

**Affiliations:** 1 *Department of Food Hygiene and Aquaculture, Faculty of Veterinary Medicine, Ferdowsi University of Mashhad, Mashhad, Iran;*; 2 *Department of Horticulture, Faculty of Agriculture, Ferdowsi University of Mashhad, Mashhad, Iran;*; 3 *Department of Clinical Sciences, Faculty of Veterinary Medicine, Ferdowsi University of Mashhad, Mashhad, Iran; *; 4 *Health Science Research Center, Department of Occupational Health Engineering, School of Health, Mashhad University of Medical Science, Mashhad, Iran.*

**Keywords:** Black zira, Essential oil, Predictive modeling, *Staphylococcus *growth

## Abstract

Black zira (*Bunium persicum*) is a medicinal plant and spice, naturally grows in Iran. The aim of this study was to investigate the combined effects of different concentrations of *Bunium persicum* essential oil (EO; including 0, 0.08, 0.16 and 0.24%), three incubation temperatures (15, 25 and 35˚C), three levels of pH (5, 6 and 7 adjusted by hydrochloric acid), and three inoculum size (10^2^, 10^3 ^and 10^4 ^CFU mL^-1^) on the growth of *Staphylococcus aureus* in the brain heart infusion broth. Black zira EO was extracted and its component was identified using gas chromatography-mass spectrometry (GC-MS) analyses. The experiment was carried out in triplicate. Growth was monitored by visible turbidity during a 30-day period. To evaluate effects of explanatory variable on time to detection (TTD) of bacterial growth, parametric survival models based on Log-normal distribution was used. All explanatory variables had significant association with time to detection (*p *< 0.05). The final model accurately predicted the growth initiation and inhibition of *S. aureus.*

## Introduction

*Staphylococcus aureus* is the most common entero-toxigenic staphylococcal species causing foodborne disease.^1^ Amongst the reported foodborne illnesses, *S. aureus* is considered the third most important cause of disease in the world.^2^ This gram-positive bacterium has no particular nutritional and environmental requirement for its growth and it can grow at a_w_ of 0.86, pH above 4.80 and its minimum growth temperature is 8.9 ˚C. Most strains are capable of producing one or more enterotoxins which are the cause of gastrointestinal symptoms observed during intoxications.^3^

As food safety is of major concern in modern society, the scientific discipline of predictive microbiology gains more and more interest worldwide. An important research topic in this field is the development of mathematical models able to predict the growth of pathogenic micro-organisms in foods. Such models present a valuable tool in risk assessment and HACCP studies.^4^

Predictive microbiology is a young discipline that has developed most of its terminology and methodology in the last two decades. It is based on the premise that the response of microorganisms (e.g., germination, growth and thermal inactivation kinetics) is reproducible, so the results from past observations can be used to predict the response of the same organism at similar environmental conditions.^5^

Essential oils (EOs) are aromatic oily liquids obtained from plant material. Terpenoids and phenolic compounds such as thymol, carvacrol and eugenol are responsible for their antimicrobial activity.^6^ Essential oils can be obtained by expression, fermentation, enfleurage or extraction, but the method of steam distillation is most commonly used for commercial production of EOs.^6^

There is considerable interest in the possible use of these compounds as food additives, to delay the onset of food spoilage or to prevent the growth of foodborne pathogens. Among these pathogens *Salmonella enteritidis *and *S. aureus* are of great importance.^7^

*Bunium persicum *[Boiss.] B. Fedtsch is an important medicinal plant and spice belonging to the Apiaceae family.The plant is growing wild in the dry temperature regions of Jammu and Kashmir, Himachal Pradesh, Afghanistan, and Iran. It is a small, grassy and perennial plant which produces white or pink flowers on the terminal and lateral stems during the third year of its life.^8^

The seeds, rich in essential oil (EO), are consumed widely as condiment. In the indigenous system of medicines, seeds are regarded as stimulants and carminatives and found to be useful in diarrhea and dyspepsia.^9^ The extracts of *B. persicum *have hypoglycemic activity and can prevent diabetes and obesity.^10^ Also, this plant is used for culinary purposes and for flavoring foods and beverages.^10^^-^^12^

This study was designed to model the combined effects of different levels of *B. persicum* EO, temperature, pH and inoculum levels on the growth of *S. aureus*.

## Materials and Methods

**Plant material. **The air-dried seeds of *B. persicum *[Boiss.] Fedtsch, were supplied from agricultural research fields of Ferdowsi University of Mashhad (FUM), Mashhad, Iran. The plant materials were authenticated in FUM herbarium and voucher specimens (No. 36267) deposited in the herbarium.

**Essential oil extraction. **Amount of 100 g of dried material were finely ground in a blender and submitted to FUM hydrodistillation facility. Hydrodistillation was done in 4 hr, using a clevenger-type apparatus. The EOs obtained were separated from water and dried over anhydrous Na_2_SO_4_, and stored in dark glass bottles at 4 ˚C prior to use.

**Gas chromatography and GC-MS analysis. **The components of the EO sample were identified by GC and GC-MS analyses. The GC-MS apparatus was a Varian GC-MS spectrometer consisting of a Varian Star 3400 GC equipped with a fused-silica column (DB-5, 30 m length × 0.25 mm inner diameter, 0.25 μm film thickness; J and W Scientific Inc., Folsom, USA), interfaced with a mass spectrometric detector (Model Varian Saturn 3; Agilent Technology Inc., Santa Clara, USA). The operating conditions were as follows: oven temperature 60-240 ˚C with a rate of 3 ˚C per min, injector temperature 280 ˚C, injector mode: split injection, with carrier gas, Helium, flow rate 2 mL min^-1^, mass spectra: electronic impact, ionization potential 70 eV, ion source temperature 250 ˚C, ionization current 1000 μA, resolution 1000 and mass range 40-300 unit. The GC was a Shimadzu GC-17 equipped with a FID detector, fused silica column (BP-5, 25 m length × 0.22 mm inner diameter, 0.25 μm film thickness). The operating conditions were: oven temperature 60-280 ˚C with a rate of 8 ˚C per min, injector temperature 280 ˚C, split ratio 1:10, with carrier gas, nitrogen, and detector temperature 300 ˚C.

**Identification of components. **The oil components were identified from their retention indices (RI) obtained with reference to the n-alkane series (Sigma, Gillingham, UK) on the DB-5 column, mass spectra with those of authentic samples, composition of their mass spectra and fragmentation patterns reported in the literature, and computer matching with MS-data bank (Saturn version 4; Agilent Technology Inc., Santa Clara, USA).^13^ Quantification of the relative amount of the individual components was performed according to the area percentage method.

**Test organism.** *Staphylococcus aureus* ATCC 25923 (Mast International Inc., Merseyside, UK) was used as the test organism in this study.

**Experimental design. **To assess the effects of *B. persicum* EO, pH, temperature, and inoculum level on growth initiation of *S. aureus*, the experiment was arranged in a factorial design in brain heart infusion broth (BHI; Merck, Darmstadt, Germany). This design (4 × 3 × 3 × 3 × 3 equal to 324 combinations) included four concentrations of EO (0, 0.08, 0.16 and 0.24%), three levels of pH (5, 6 and 7) adjusted by hydrochloric acid, three incubation temperatures (15, 25 and 35 ˚C), three inoculums size (10^2^, 10^3 ^and 10^4 ^CFU mL^-1^), three replicate of all combinations and repeated observations (daily) for growth in BHI broth for up to 30 days.

**Preparation** **o** **f inoculum. **The reference bacterium was plated on Baired Parker agar (Merck, Darmstadt, Germany) agar medium and incubated at 37 ˚C for 24 hr. After confirmation typical colonies as *S. aureus* by bio-chemical tests, inoculums were prepared by transferring a loop full of the bacterial colonies to isotonic saline solution in a sterile cuvette to adjust the absorbance of 0.02 at 600 nm using a spectrophotometer (Model 6105; Jenway, Essex, UK). This adjustment gave a cell concentration of 1.2 × 10^9 ^CFU mL^-1^. The numbers of cells in the suspension were estimated by duplicate plating from 10-fold serial dilutions on BHI agar and counting the colonies after 24 hr incubation at 37 ˚C.

**Performing the experiment. **Amount of 3.7 g BHI broth powder (Merck, Darmstadt, Germany) was dissolved in 90 mL distilled water in a 250 mL flask by mild heating. In order to produce and maintain a stable oil-water emulsion in broth substrate during the period of study (30 days), the method explained by Mann and Markham were used,^14^ with some modifications. Briefly, we added 5% (v/v) dimethyl-sulfoxide (Merck, Darmstadt, Germany) as an emulsifier and 0.05% (w/v) agar (Merck, Darmstadt, Germany) as a stabilizer to the broth substrate. The pH was adjusted using hydrochloric acid to designate pH values. The values of pH were adjusted using a pH meter (Jenway, Staffordshire, UK). The final volume of broth substrate was brought to 100 mL with additional distilled water. The content of each flask was autoclaved at 121 ˚C for 15 min. After cooling, the pH of each combination in broth medium was measured and adjusted again to the considered pH using 1M filter sterilized HCl (or NaOH). Then filter sterilized EO was added in different amounts to satisfy the experimental design. The content of flask containing sterile BHI broth was dispensed in portions of 3 mL into 16 × 100 mm sterile caped tubes (Becton Dickinson, San Jose, USA). The tubes were inoculated with *S. aureus* culture (10^2^, 10^3^ and 10^4 ^CFU mL^-1^). For each combination the inoculated tubes were stored at 15, 25 and 35 ˚C for up to 30 days. During these periods all the tubes were observed for visible growth (turbidity) daily up to 30-days. The number of tubes (combinations) showing growth at a particular observation were recorded. For each combination a negative control (un-inoculated tube) was used. All experiments were conducted in independent triplicate.

**Statistical Analysis. **The time to visible growth (TTD: time-to-detection or time to the nearest visible growth detection) considered as outcome variable in the present study. Since some combinations did not grow until the end of the study (30 days), event-time (survival) analysis was employed.

Kaplan-Meier survival curves for each level of an explanatory variable were plotted and the homogeneity of the curves between levels tested using the log rank statistic. Explanatory variables that showed a significant association with TTD were selected for inclusion in the multivariate analysis.

Parametric survival model based on accelerated failure time (AFT) approach^15^ was used to quantify the effect of each of the prescribed explanatory variables on time to detection of bacterial growth. The general form of the accelerated failure time model is:

*log(t) = (α + β*_1_ *x*_1i_ *+ … + β*_m_ *x*_mi_ *) + log(* *τ)*

where *log(t)* is the natural logarithm of the time to ‘failure’ (growth),* α* an intercept term, *β*_1_*x*_1i_ + … + *β*_m_*x*_mi_ is a linear combination of the *m* explanatory variables and their regression coefficients, and *log(τ)* is an error term. Using this approach the accelerated failure time coefficients represent the expected change in *log(t)* for changes in the predictor levels. 

In the present study the fit of the exponential, weibull, log-normal and log-logistic distribution to the current data was evaluated using mean square error (MSE) values as below. The smaller MSE value indicates a better fit.

$\mathrm{MSE}=\frac{\sum {\left(\mathrm{predicted}-\mathrm{observed}\right)}^{2}}{n-p}$

where *n* is the number of observations and *p* is the number of parameters to be estimated.

To select those explanatory variables that best explained time to detection a backward stepwise approach was used. Explanatory variables that were not statistically significant were removed from the model one at a time, beginning with the least significant, until the estimated regression coefficients for all retained variables were significant at *p *< 0.05. All analyses were carried out using Stata Statistical Software, version 10 (Stata-Corp, College Station, Texas, USA).

## Results

**Chemical composition of** ***B. Persicum*** **Boiss. EO. **The components of essential are presented in Table 1. The yield of oil from *B. persicum *was 9.10% (v/w). GC/MS identified 35 compounds, representing 95.50% of the oil content. γ-terpinene was the main monoterpene hydrocarbon, with a content of 44.20%. The cumin-aldehyde and ρ-cymene contents determined as 16.90 and 8.00%, respectively. 

**Description of growth and no growth. **About 84.75% of combinations (270 out of 324) showed growth during the study period and 15.25% of combinations (54 out of 324) did not grow and were considered as censored observations.

**Table 1:** Phytochemical composition of *B. persicum* essential oil

**Phytochemicals**	**RI** a	**Percentage**	
**α** **-Thujene**	925		0.4
**α** **-Pinene**	932		1.0
**Camphene**	946		0.1
**Sabinene**	970		1.2
**ß-Pinene**	975		1.6
**Myrcene**	990		1.0
**δ** **-2-Carene**	1002		tr^b^
**Isosylvestrene**	1013		0.3
**ρ** **-Cymene**	1019		8.0
**Limonene**	1025		2.0
**1,8-Cineole**	1032		2.9
**Z-ß-Ocimene**	1037		0.1
**γ** **-Terpinene**	1055		44.2
**3-methylbenzaldehyde**	1059		tr
**cis-Sabinene hydrate**	1061		tr
**Terpinolene**	1085		0.7
**Linalool**	1093		0.1
**trans-Sabinene hydrate**	1095		0.1
**Borneol**	1162		0.1
**Terpinen-4-ol**	1170		0.4
**α** **-Terpineol**	1189		tr
**meta-Cuminol**	1217		tr
**p-Cuminaldehyde**	1231		16.9
**trans-p-Menth-2-en-7-ol**	1261		0.2
**Perillaldehyde**	1265		0.2
**Bornyl acetate**	1280		2.9
**α** **-Terpinen-7-al**	1281		0.4
**γ** **-Terpinen-7-al**	1287		10.5
**Thymol**	1289		0.1
**9-epi-ß-Caryophyllene**	1413		tr
**ar-Curcumene**	1474		tr
**Germacrene D**	1476		0.1
**α** **-Zingiberene**	1490		tr
**EE-** **α** **-Farnesene**	1503		tr
**ß-Sesquiphellandrene**	1518		0.1
**Total identified**		95.5	

a Retention index relative to n-alkane series on the DB-5 column.

b Trace (< 0.0 5%)

**Fig. 1:**
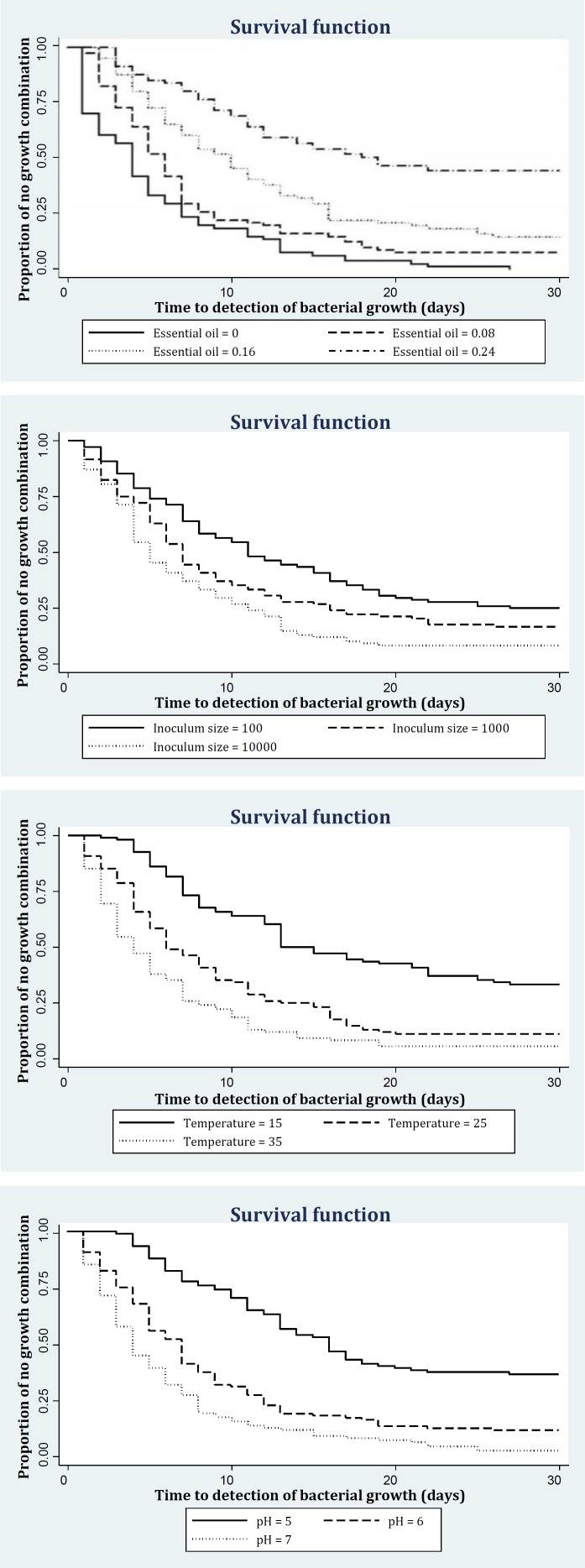
Kaplan-Meier survival curves showing the proportion of no growth combinations for different levels of essential oil, inoculum size, temperature and pH.

**Evaluation of time to detection of bacterial growth. **Median time to detection of bacterial growth was 7 days. Kaplan-Meier survival curve for different levels of explanatory variables are presented in Figure 1.

On the basis of MSE value the log normal model provided the best fit to the data. The MSE value of the log normal model was 207.97. While the MSE values were 211.27, 250.10 and 584.70 for log-logistic, Weibull and exponential models, respectively. The final model showed that all explanatory variables had significant (*p *< 0.001) association with time to detection, (Table 2).

On average, time to detection for combinations with 0.08%, 0.16% and 0.24% of *B. persicum* essential oil was 1.72, 2.92 and 5.49 times greater than those without it, respectively. Time to detection (TTD) for those combinations with pH levels of 6 and 5 was 1.53, and 4.28 times greater than those with pH level of 7. Also, this time for combinations with inoculum level of 10^3 ^and 10^2^ was 1.38 and 2.15 times greater than combinations with inoculum level of 10^4^. Furthermore, this period for combinations with incubation temperature of 25 ˚C and 15 ˚C was 1.59 and 3.95 times greater than combinations with those by incubation temperature of 35 ˚C.

The final model equation is shown below:

_TTD =_ *e – 0.361 + 1.37T15 0.463T25 + 0.765IL102 + 0.327IL103 + 1.45pH5 + 0.424pH6 + 1.7 EO0.24 + 1.07 EO0.16 + 0.547 EO0.08*

where *TTD* is time to detection, *e* is a mathematical constant approximately equal to 2.718281828, *T* is temperature, *IL* is inoculums size, and *EO* is essential oil.

**Fig. 2:**
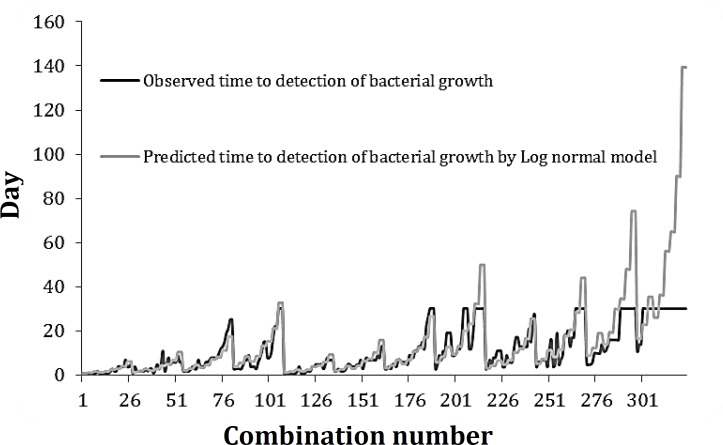
Observed and predicted days needed for growth initiation of *S*. *aureus* (TTD) according to log normal model

The model predicts the value of TTD describing the growth of *S. aureus*, as environmental factors change. From these models the values of predicted TTD can be calculated from any combination of EO, T, pH and IL with the limits studied. Observed and predicted time to detection of bacterial growth for each combination presented in Figure 2.

**Table 2:** Accelerated failure time model of factors influencing time to detection of bacterial growth

**Variable**	**β(SE)**	***p*** **-value**	**Time ratio (95% CI)**
**Intercept**	-0.361(0.068)	< 0.001	
**Essential oil**			
**0**	0		1
**0.08**	0.547(0.058)	< 0.001	1.72 (1.54-1.94)
**0.16**	1.07(0.059)	< 0.001	2.92 (2.60-3.28)
**0.24**	1.70(0.063)	< 0.001	5.49 (4.85-6.23)
**pH**			
**7**	0		1
**6**	0.424(0.051)	< 0.001	1.53 (1.38-1.70)
**5**	1.45(0.055)	< 0.001	4.28 (3.84-4.77)
**Inoculum level**			
**10** ^4^	0		1
**10** ^3^	0.327(0.053)	< 0.001	1.38 (1.25-1.54)
**10** ^2^	0.765(0.052)	< 0.001	2.15 (1.94-2.39)
**Temperature**			
**35˚ C**	0		1
**25˚ C**	0.463(0.051)	< 0.001	1.59 (1.44-1.75)
**15˚ C**	1.37 (0.054)	< 0.001	3.95 (3.55-4.40)
**sigma**	0.369 (0.016)		

## Discussion

Predictive microbiology combines mathematical modeling with experimental data on combinations of factors that influence the growth of food spoilage and/or food-borne pathogenic microorganisms. The developed models are intended to predict the fate of micro-organisms in foods.^16^

In this study the effect of *B. persicum* EO, pH, temperature and inoculum level have been evaluated on the growth responses of *S. aureus* in BHI broth and the log normal model proposed to predict these growth responses. 

Plant EOs are potentially useful sources of antimicrobial compounds.^17^ *In vitro* studies have demonstrated antibacterial activity of EOs against different bacteria at levels between 0.2-10 µL mL^-1^. Essential oils containing phenolic compounds, e.g. thymol, carvacrol, terpinene and p-cymene, are widely reported to possess high levels of antibacterial activity and may have applications in controlling pathogens in food.^6^^,^^18^^,^^21^

Despite the strong antimicrobial activity of EOs, their practical application is currently limited due to their undesirable flavor changes they cause in food products.^22^^,^^23^

The results of the present study showed that the major compound of *B. persicum *EO was phenolic monoterpene γ- terpinene (44.20%). Other important compounds were cuminaldehyde (16.90%) and ρ-cymene (8.00%).

Several researchers have investigated antibacterial and antifungal effect of *B. persicum* EO.^20^^,^^24^^,^^27^ It has been proposed that the antifungal activity of *B. persicum* EO is due to cuminaldehade.^25 ^Previous studies have shown that *B. persicum* essential oil had strong activity against *S. aureus.*^24^^,^^26^^,^^27^

In the present study on the basis of MSE value, the log normal model provided the best fit to the data. We used the concepts of the TTD in a factorial design study to quantify the effect of *B. persicum* EO, pH, and temperature and inoculum level on the growth responses of *S. aureus *in BHI broth medium. Our results indicated that in the final model all explanatory variables had significant (*p *< 0.05) association with time to detection. 

To sum up, evidence provided in this study showed that the values of TTD were higher at low levels of temperature, pH, and inoculums but high level of EO. The same results have been reported by Valero *et al*. and Jamshidi *et al*.^28^^,^^29^ In agreement to results of Goudarzi *et al*. and Barros *et al*., the present study showed that the anti-microbial properties of plant EO are dose-dependent.^30^^,^^31^ According to our results, increasing the *B. persicum* EO concentration had a significant effect (*p *< 0.001) on time to detection of *S. aureus*. By increasing the concentration of EO, the growth initiation of organism and also proportion of no growth combinations increased.

Temperature is the most common hurdles used to control microbial growth. According to our results, decreasing the incubation temperature had a significant effect on growth initiation of inoculated bacteria. Probably the most important effect of temperature on growth of a micro-organism is on the shape of enzymes required for metabolism and they will have the proper shape only within a relatively narrow range of temperatures or it may be due to the lower metabolic activity at the lower temperature.

Tassou *et al*. showed the effect of temperature and different concentrations (1.2% v/v) of mint EO on the growth/survival of *S. aureus* in nutrient broth. Their results showed that both factors significantly affected the detection time.^7^

Basti *et al*. studied the effects of *Zataria multiflora* Boiss. EO, pH and temperature on *S. Typhimurium* and *S. aureus*.^19^ They showed that TTD of both organisms were significantly affected by temperature, EO and pH, (*p *< 0.01).

The growth and metabolism of microorganisms are influenced by pH, because acidity or alkalinity of an environment has a profound effect on the activity and stability of macromolecules.^32^

According to our results the growth of *S. aureus* was significantly affected by pH. When the pH was decreased, the growth initiation of *S. aureus* and proportion of no growth combinations were increased. This can be attributed either the direct effect of pH or to the better dissolving of the EO in the lipid phase of the bacterial membrane at the low pH.^33^ In agreement to these results Basti *et al*. have shown the inhibitory action of *Zataria multiﬂora *Boiss. EO on the growth initiation of *S. Typhimurium* and *S. aureus* was enhanced by decreasing the pH value at each deﬁned temperature.^19^

In this study we used three levels of inoculation. According to our results the growth of *S. aureus* was affected significantly by the inoculum size. Our results showed that the TTD for combinations with inoculum level of 10^3 ^and 10^2^ was 1.38 and 2.15 times greater than combinations with inoculum level of 10^4^. Several studies have indicated the importance of inoculum size on the ability of a microbial population to initiate growth.^34^^-^^37^

Skandamis *et al*. have modeled the effect of inoculum size and acid adaptation on growth/no growth interface of *E. coli* O157:H7.^38^ They found that regarding the effect of inoculum concentration, the lower was the initial population, the higher were the pH levels allowing growth, especially at low temperatures (i.e., 10 and 15 ˚C).

Zhao *et al*. developed linear regression model with polynomial terms for analyzing the effect of environmental factors on time to detection. Their results showed when temperature was increased, the TTD was increased and when the pH and inoculums size were increased, the TTD was decreased. They also described a very good correlation of predicted and observed values of TTD.^39^ This was in agreement with our results were obtained for the effects of the designated factors on the TTD of *S. aureus* within the studied limits.

The results showed the observed time to detection of bacterial growth and value that predicted by log normal model equation for TTD of *S*. *aureus* in designated combinations. The graph demonstrated good agreement between predicted and observed values. So our model adequately predicted the growth initiation, and inhibition conditions of *S*. *aureus* as affected by EO, pH, temperature, and inoculum level. 

The predicted values may not match with whatever would occur in any special food system. This means that before the models could be used in such a manner, the user would have to validate the models for each specific food of interest.
